# Expression of serotonin receptors in human lower esophageal sphincter

**DOI:** 10.3892/etm.2014.2050

**Published:** 2014-11-04

**Authors:** HE-FEI LI, JUN-FENG LIU, KE ZHANG, YONG FENG

**Affiliations:** Department of Thoracic Surgery, Fourth Hospital of Hebei Medical University, Shijiazhuang, Hebei 050011, P.R. China

**Keywords:** serotonin receptors, human, lower esophageal sphincter, sling fibers, clasp fibers

## Abstract

Serotonin (5-HT) is a neurotransmitter and vasoactive amine that is involved in the regulation of a large number of physiological functions. The wide variety of 5-HT-mediated functions is due to the existence of different classes of serotonergic receptors in the mammalian gastrointestinal tract and nervous system. The aim of this study was to explore the expression of multiple types of 5-HT receptor (5-HT_1A_R, 5-HT_2A_R, 5-HT_3A_R, 5-HT_4_R, 5-HT_5A_R, 5-HT_6_R and 5-HT_7_R) in sling and clasp fibers from the human lower esophageal sphincter (LES). Muscle strips of sling and clasp fibers from the LES were obtained from patients undergoing esophagogastrectomy, and circular muscle strips from the esophagus and stomach were used as controls. Reverse transcription-polymerase chain reaction (RT-PCR), quantitative PCR and western blotting were used to investigate the expression of the various 5-HT receptor types. Messenger RNA for all seven 5-HT receptor types was identified in the sling and clasp fibers of the LES. At the mRNA level, the expression levels were highest for 5-HT_3A_R and 5-HT_4_R, and lowest for 5-HT_5A_R, 5-HT_6_R and 5-HT_7_R. At the protein level, the expression levels were highest for 5-HT_3A_R and 5-HT_4_R, followed by 5-HT_1A_R and 5-HT_2A_R; 5-HT_7_R was also detected at a low level. The expression of 5-HT_5A_R and 5-HT_6_R proteins was not confirmed. The results indicate that a variety of 5-HT receptor types can be detected in the human LES and probably contribute to LES function.

## Introduction

Serotonin (5-hydroxytryptamine, 5-HT) has important biological functions that are mediated via 5-HT receptors. Seven families of 5-HT receptors, designated from 5-HT1 to 5-HT7, are currently recognized and more than sixteen subtypes have been identified in humans. With the exception of the 5-HT3 receptor, the receptors are members of the seven transmembrane domain G protein-coupled receptor family, while the 5-HT3 receptor is a ligand-gated ion channel belonging to the Cys-loop superfamily of pentameric proteins (1).

The majority of 5-HT in the body is produced by enterochromaffin cells in the gut. 5-HT receptors are widely distributed in the gastrointestinal mucosa and muscle layers, and play an important role in the functional mediation of the gastrointestinal tract (2). It has been found that 5-HT receptors are widely expressed in the gastrointestinal tracts of mammals, such as the rat and opossum (3). However, there is little information available concerning 5-HT receptor expression in the human lower esophageal sphincter (LES). The LES is an important physiological structure at the esophagogastric junction. Abnormalities in the LES are closely associated with dysfunction in gastrointestinal motility disorders such as achalasia and gastroesophageal reflux disease (GERD) (4).

The objective of the present study was to detect 5-HT receptors in the human LES, in particular, within the clasp and sling fibers of the LES. Following the identification of their expression patterns, the role of the 5-HT receptors in the modulation of human LES function was further investigated.

## Materials and methods

### Patients and tissue retrieval

The experimental protocol has been approved by the Research Ethics Committee of the Fourth Hospital of Hebei Medical University (Shijiazhuang, China). Written informed consent was obtained from the patients. Muscle strips were collected from 28 patients who underwent esophagectomy for mid-third esophageal carcinoma in the Department of Thoracic Surgery at this hospital from March 2012 to August 2012. There were 16 males and 12 females, with an average age of 58 years (range, 50 to 67 years). Patients with a history of GERD, achalasia, scleroderma, or other diseases associated with a disorder of the LES were excluded from the study. Each specimen was resected en bloc in the operating room, and the fresh specimen was placed immediately in ice-cold Krebs solution. The Krebs solution had the following composition (in mM): sodium, 143.0; potassium, 5.0; calcium, 2.5; magnesium, 1.2; chloride, 128.0; phosphate, 2.2; bicarbonate, 24.9; sulfate, 1.2; and glucose, 10.0. Specimens were not included in this study if any segment that was required for study contained a macroscopically visible tumor.

In the laboratory, the fresh specimens of the gastroesophageal junction were opened along the long axis of the esophagus and the greater curvature of the stomach. Specimens were pinned to a wax plate in the presence of Krebs solution at 37°C to maintain its approximate *in situ* dimensions, with a continuous supply of mixed gas of 95% O_2_ and 5% CO_2_. The mucosa and submucosa were then removed by dissection.

The sling and clasp fibers were identified as thickened bands of circular oriented smooth muscle in the gastric cardia, adjacent to the greater and lesser curvature of the stomach, respectively. The sling and clasp muscle strips were prepared using a previously described method (5,6). In addition, circular muscle fibers from the esophagus above the LES, and circular muscle fibers from the gastric fundus below the LES, were dissected for use as control specimens. These circular muscle strips were obtained 3 cm proximal and distal to the gastro-esophageal junction. All specimens were removed carefully to ensure that the myenteric plexus and the longitudinal muscle in the wall of the esophagus and stomach were excluded. The dissected muscle strips from the four parts were frozen in liquid nitrogen and stored at −80°C for subsequent RNA extraction.

### RNA isolation and reverse transcription-polymerase chain reaction (RT-PCR) for 5-HT receptor analysis

Tissue was homogenized in TRIzol reagent (Invitrogen Life Technologies, Carlsbad, CA, USA) at a ratio of 100 mg tissue to 1 ml TRIzol, and then centrifuged at 12,000 × g for 5 min. Total RNA was extracted by acid guanidinium thiocyanate-phenol-chloroform extraction. The quality of the RNA was verified by agarose gel electrophoresis using ethidium bromide staining. First-strand cDNA synthesis (reaction volume, 20 μl) using 2 mg RNA was performed by RT reaction in the presence of RevertAid Moloney Murine Leukemia Virus (M-MuLV) reverse transcriptase (Fermentas, Thermo Fisher Scientific, Waltham, MA, USA). RT was conducted with 0.5 mg oligo(dT)_18_, using diethypyrocarbonate (DEPC)-treated water to achieve a volume of 11 μl, and the reaction mixture was incubated at 70°C for 5 min, prior to being chilled on ice. Next, 4 μl 5X reaction buffer (Fermentas), 2 μl 10 mM 4 dNTP mix and 20 units RNasin (both from Fermentas) were added, using DEPC-treated water to provide a reaction volume of 19 μl, and the mixture was incubated at 37°C for 5 min. Finally, 200 units (1 μl) RevertAid M-MuLV reverse transcriptase was added, and the reaction mixture was incubated at 37°C for 50 min, before the reaction was stopped and held at 70°C for 15 min.

PCR amplification of the cDNA was performed using primers designed specifically to match the 5-HT receptor mRNA (primers listed in Table I). In each PCR reaction, 2 μl cDNA reaction mixture was used. PCR was performed in a 20-μl reaction volume. The amplification conditions were: 5 min initial denaturation at 95°C, then 36 cycles of 95°C for 35 sec, 60°C for 35 sec and 72°C for 45 sec followed by 72°C for 5 min. A negative control in which all the components of the reaction were added, except the cDNA template was tested in parallel with each sample to identify any risk of false positive results. The amplified products were electrophoresed on a 1.5% agarose gel and PCR products were determined using the Gel-Pro gel image analysis system (Media Cybernetics, Silver Spring, Maryland, USA). Densitometry for analysis of the PCR product bands was conducted by the imaging software. The relative expression level of each gene was normalized by the value of β-actin. The amplified products were analyzed by electrophoresis on 1.5% agarose gels and visualized by ethidium bromide staining, with images captured by photography under a UV transilluminator. The integrated optical density (IOD) of the gel was calculated with Gel-Pro software (Media Cybernetics). The relative expression level of the mRNA of each 5-HT receptor type was normalized by the value of β-actin.

### Quantification by quantitative PCR (qPCR)

The qPCR experiments were conducted using an Applied Biosystem 7500 Real-Time PCR System (Applied Biosystems, Foster City, CA, USA) and data were analyzed with ABI 7500 (version 2.0.6) software. All oligonucleotide primers for qPCR were designed using Primer 3 software (http://frodo.wi.mit.edu/cgi-bin/primer3/primer3www.cgi) and synthesized by Invitrogen Life Technologies. The diluted cDNA (1 μl from each sample) was used as PCR template. The reaction composition contained: 1 μl diluted cDNA; 12.5 μl 2X TransStart Top Green qPCR SuperMix (TransGen Biotech, Beijing, China.), 10 μl ddH_2_O, 0.5 μl forward primer and 0.5 μl reverse primer, and 0.5 μl Passive Reference Dye (TransGen Biotech) in a final volume of 25 μl. The cDNA was amplified by one cycle at 94°C for 30 sec, followed by 42 cycles of 95°C for 5 sec, 60°C for 34 sec and melt curve analysis. Reactions were performed in triplicate according to the manufacturers’ instructions (TransGen Biotech). Melting point and melting curve analyses were undertaken on each set of reactions to confirm that only a single product was produced. No primer-dimers were detected by melting point analysis and this was confirmed in preliminary runs with gel electrophoresis. The expression level of the target gene was calculated by the 2^−ΔΔCt^ method (7). The relative expression of target gene mRNA was indexed to the reference gene β-actin using the formula: 10,000 × 2^ΔCt^, in which ΔCt = Ct_β-actin_ - Ct_target gene_.

### Western blot analysis of 5-HT receptors

Total proteins were extracted from the muscle strips using a protein extraction kit (Solarbio, Beijing, China). Protein concentration was determined using a colorimetric bicinchoninic acid (BCA) protein assay reagent (Multisciences, Hangzhou, China). Following denaturation at 100°C for 10 min, aliquots of protein samples (30 μg) were separated by electrophoresis on SDS-polyacrylamide gel 10% separation gel and 4% pycnotic gel, separated at 150 V for 1 h, and transferred onto a polyvinylidene difluoride (PVDF) membrane, which was then blocked for 1 h with 5% non-fat milk in Tris-buffered saline with Tween 20 (TBST) at room temperature, and incubated with an anti-human, polyclonal primary antibody (dilutions: rabbit anti-5HT_1A_R, 1:300; rabbit anti-5HT_2A_R, 1:400; rabbit anti-5HT_3A_R, 1:500; rabbit anti-5HT_4_R, 1:500; rabbit anti-5HT_5A_R, 1:300; rabbit anti-5HT_6_R, 1:300; rabbit anti-5HT_7_R, 1:300; 1:10,000 for the rabbit anti-β-actin; Abcam Trading, Shanghai, China) at 4°C overnight. After washing three times with TBST at room temperature for 30 min in total, the membrane was incubated with a goat anti-rabbit IgG polyclonal secondary antibody (1:2,000, anti-rabbit IgG; Abcam Trading) for 1 h. After three washes with TBST, the membrane was analyzed using an infrared fluorescence imaging instrument (Odyssey Infrared Imaging System, American LI-COR, Lincoln, Nebraska, USA). The IOD value was calculated by the Gel-Pro software and the relative expression level of each protein was normalized by the value of β-actin.

### Statistical analysis

Results are expressed as the mean ± standard deviation (SD). SAS software, version 9.2 (SAS Institute Inc., Cary, NC, USA) was used to conduct the statistical analysis. Differences in the mRNA and protein expression levels were analyzed with one-way analysis of variance, and the Student-Newman-Keuls multiple range (SNK-q) test was used to evaluate comparisons within groups. P<0.05 was considered to indicate a statistically significant difference.

## Results

### Characterization of mRNA encoding 5-HT receptors

**Figure 1:**
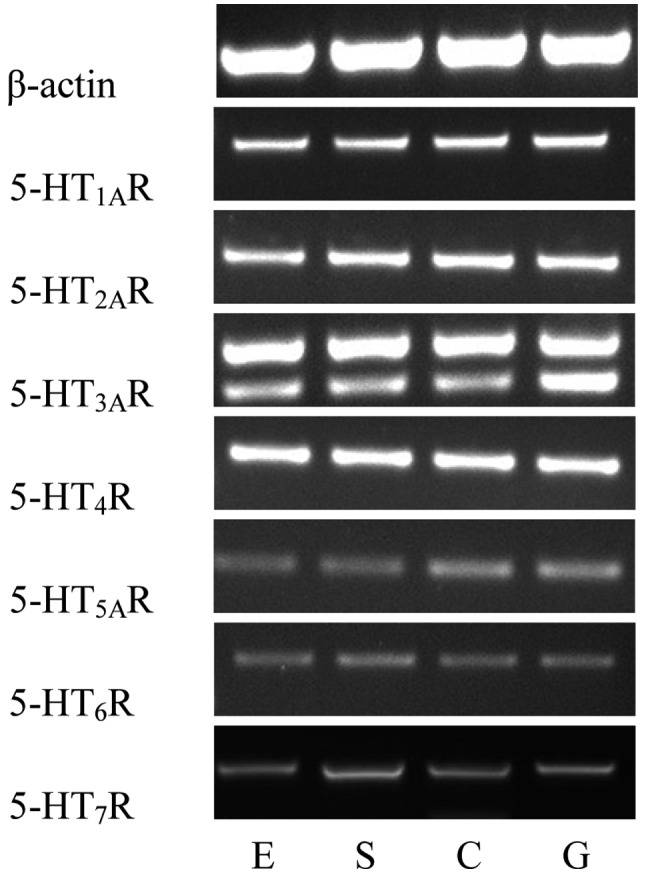
mRNA expression of 5-HT receptors in the sling and clasp fibers of the LES and circular muscle strips from the esophagus and stomach. A representative example of the RT-PCR products specific for each 5-HT receptor mRNA is shown. 5-HT, serotonin; LES, lower esophageal sphincter; RT-PCR, reverse transcription-polymerase chain reaction; E, circular muscle strip of esophagus; S, sling fibers; C, clasp fibers; G, stomach.

Using RT-PCR, the mRNA expression levels of seven 5-HT receptors in the human LES were determined. Distinct bands of the expected sizes were detected for each of the seven 5-HT receptor mRNAs and their levels of expression appeared to differ. Similar results were obtained in all PCR assays performed on mRNA extracted from the 28 patients. The primer pairs designed to recognize the 5-HT_3A_R and 5-HT_4_R mRNA generated strong bands, indicating high expression levels of the 5-HT_3A_R and 5-HT_4_R mRNA. The 5-HT_1A_R and 5-HT_2A_R mRNA also generated comparatively strong bands. However, the primer pairs for the 5-HT_7_R, 5-HT_5A_R and 5-HT_6_R mRNA produced relatively weak bands (Fig. 1).

### Quantification of 5-HT receptor mRNA expression

**Figure 2:**
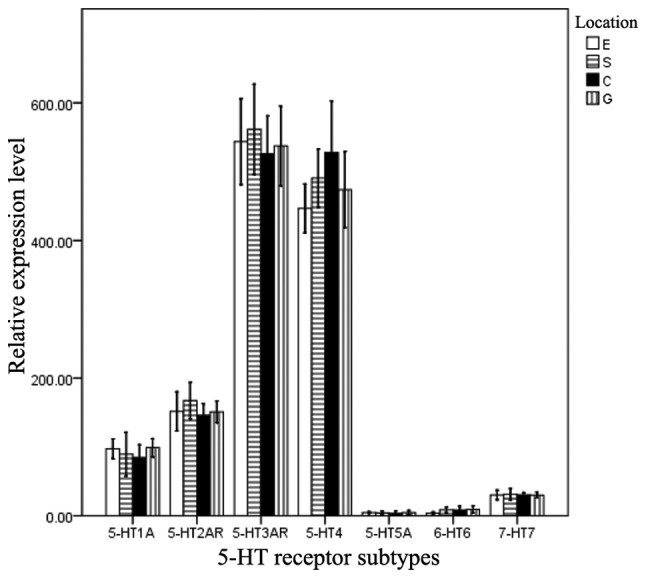
Quantitative determination of the mRNA expression levels of various 5-HT receptors in the sling and clasp fibers of the LES and circular muscle strips from the esophagus and stomach. Bar chart showing the relative mRNA expression levels of the various 5-HT receptors normalized against β-actin. There were significant differences between the 5-HT receptor subtypes in the same muscle strip (P<0.05), but no significant differences for each subtype across various muscle strips (P>0.05). 5-HT, serotonin; LES, lower esophageal sphincter; IOD, integrated optical density; E, circular muscle strips of esophagus; S, sling fibers; C, clasp fibers; G, stomach.

To compare the expression levels of the seven different 5-HT receptor mRNAs, qPCR was performed. Significant differences were identified when the mRNA expression levels of the 5-HT receptors were compared in the same muscle strip (F, 78.281; P=0.000). The rank order of expression was as follows: 5-HT_3A_R = 5-HT_4_R > 5-HT_1A_R = 5-HT_2A_R > 5-HT_5A_R = 5-HT_6_R = 5-HT_7_R. However, no significant difference was observed in the mRNA expression levels of the 5-HT receptors among the four types of muscle strip (F, 0.232; P=0.731; Fig. 2).

### Expression of 5-HT receptor proteins

**Figure 3:**
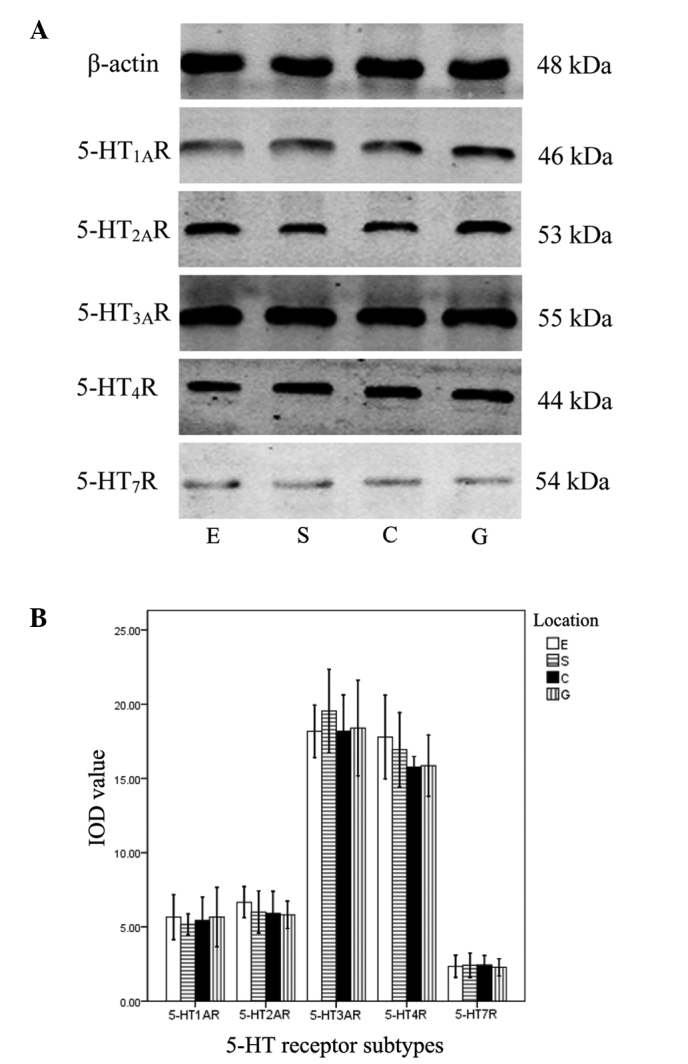
Expression of 5-HT receptor subtypes determined by western blot analysis in the sling and clasp fibers of the LES and circular muscle strips of the esophagus and stomach. (A) Bands of five 5-HT receptor subtypes in E, S, C and G were identified by western blotting. (B) IOD values of the bands. There were significant differences between the 5-HT subtypes in the same muscle strip (P<0.05), but no significant differences were identified in a single subtype between the four types of muscle strip (P>0.05). 5-HT, serotonin; LES, lower esophageal sphincter; IOD, integrated optical density; E, circular muscle strips of esophagus; S, sling fibers; C, clasp fibers; G, stomach.

With the exception of 5-HT_5A_R and 5-HT_6_R proteins, the other five receptors were identified. Significant differences in the IOD values for the different 5-HT receptors in the same muscle strip were observed (F, 657.357; P=0.000). The rank order of the IOD values was as follows: 5-HT_3A_R = 5-HT_4_R > 5-HT_1A_R = 5-HT_2A_R > 5-HT_7_R. No significant differences in IOD values were identified among the four type of muscle strip (F, 0.194; P=0.801; Fig. 3).

## Discussion

Serotonin (5-HT), as a predominant neurotransmitter, controls a variety of functions, including locomotor activity, cognition, emotion, food intake and endocrine regulation, via effects on 5-HT receptors. Currently, seven 5-HT receptors and more than sixteen 5-HT receptor subtypes have been identified as members of the G protein-coupled receptor or ligand-gated ion channel families. More specifically, these are the receptor subtypes of 5-HT1A, 5-HT1B, 5-HT1C, 5-HT1D, 5-HT1E, 5-HT1F, 5-HT2A, 5-HT2B, 5-HT2C, 5-HT3A, 5-HT3B, 5-HT4, 5-HT5A, 5-HT5B, 5-HT6 and 5-HT7, of which the 5-HT7 receptor is divided into 5-HT7(a), 5-HT7(b) and 5-HT7(c) in humans. The 5-HT receptors are widely distributed in the central and peripheral nervous system, cardiovascular system and gastrointestinal tract in mammals, and have been shown to play significant physiological roles (8–10). Various esophageal motility disorders, such as achalasia, diffuse esophageal spasm and nutcracker esophagus, are associated with motor dysfunction of the LES (11). The regulatory mechanism of the LES involves various receptors (12), neurotransmitters and signal transduction pathways., including CCK, muscarinic and dopamine receptors (5,6,13).

5-HT receptors are widely distributed in smooth muscle in various parts of the body, including the gastrointestinal tract, where they control aspects of gastrointestinal motility and secretion (14). 5-HT and serotonergic agonists and antagonists have been found to exert pharmacological effects on various regions of the gut. For example, 5-HT_3_ receptor antagonists have antiemetic activity and 5-HT_4_ receptor agonists are used to promote gastrointestinal peristalsis in the clinic (15,16). Previous studies have evaluated the distribution of 5-HT receptors in the gastrointestinal tract. Mader *et al* (17) found that 5-HT_4_ receptor mRNA expression was present throughout the gastrointestinal tract in humans and primates, which supports findings that 5-HT_4_ receptors exhibit multiple effects in the gastrointestinal system. Champaneria *et al* (18) identified the expression of the 5-HT_3_ receptor in rat gut. Irving *et al* (19) evaluated 5-HT_3_, 5-HT_4_ and 5-HT_7_ receptor expression and compared 5-HT_4_ and 5-HT_7_ receptor function in the circular muscle of the human colon. In addition, the 5-HT_1A_ receptor was identified in guinea pig and human intestine by Wang *et al* (20). These findings prompt the hypothesis that 5-HT receptors are located throughout the gastrointestinal tract. However, prior to the present study, little information was available concerning the distribution of 5-HT receptors in the LES. The present study was designed to determine whether 5-HT receptors exist in the region of the human LES. The LES is a complex structure comprising clasp and sling fiber muscle strips in the gastric cardia, and circular muscle fibers in the distal end of the esophagus immediately above the gastroesophageal junction. In the present study, the clasp and sling fiber muscle strip component of the LES was investigated. 5-HT_5_ and 5-HT_6_ receptors mainly exist in the central nervous system and are associated with memory, mood, pain and cognition (21–25). The gastrointestinal tract is known to express 5-HT_1_, 5-HT_2_, 5-HT_3_, 5-HT_4_ and 5-HT_7_ receptors, which regulate the function of gastrointestinal tract. The 5-HT_1_ and 5-HT_7_ receptors are considered to be relaxant and the 5-HT_2_, 5-HT_3_ and 5-HT_4_ receptors to be contractile (26–30).

The selective 5-HT_1A_ receptor agonists sumatriptan and buspirone have been identified to enhance esophageal peristalsis and LES function (31). Cohen *et al* (32) found that 5-HT induced contractions mediated by the 5-HT_2_ receptor in guinea pig and rabbit esophagus. In the present study, the presence of the 5-HT_2_ receptor in distal esophageal muscle and in the human LES was clearly identified. Among all 5-HT receptors, the 5-HT_3_ and 5-HT_4_ receptors have been the most thoroughly studied in the gastrointestinal system. 5-HT_3_ receptors mainly exist in the nervous system and gastrointestinal tract; the most well established physiological roles of the 5-HT_3_ receptor are in the coordination of emesis and regulation of gastrointestinal motility (33). 5-HT_3_ receptor antagonists such as ondansetron and granisetron can mediate gastrointestinal contraction and intestinal secretion, and antagonize 5-HT-induced relaxation of the esophagus by increasing LES tone (34). The 5-HT_4_ receptor-mediated response is predominant in the 5-HT-induced acceleration of motility associated with acetylcholine release in the gastrointestinal tract (35,36), and previous studies have identified the 5-HT_4_ receptor is localized on the myenteric plexus (37). The 5-HT_7_ receptor was the last member of 5-HT receptor family to be discovered (35,38). 5-HT_7_ receptors have been implicated in the pathophysiology of several disorders; they play a role in smooth muscle relaxation within the vasculature and in the gastrointestinal tract (39,40). Liu *et al* (41) identified the expression of four subtypes of 5-HT_7_ receptor throughout the rat gastrointestinal tract. Yang *et al* (42) concluded that a 5-HT signaling pathway disorder may be a major factor in the pathogenesis of gastroesophageal reflux and reflux esophagitis in experiments on rats.

Through the use of RT-PCR and qPCR, the present study identified the mRNA of all seven 5-HT receptors in human LES sling fibers, clasp fibers, circular muscles of the esophageal body and gastric fundus. 5-HT_3A_R and 5-HT_4_R expression levels were the highest, followed by those of 5-HT_1A_R and 5-HT_2A_R; the lowest expression levels were found for 5-HT_5A_R, 5-HT_6_R and 5-HT_7_R. Western blotting confirmed the expression of five of the 5-HT receptors, with the exception of 5-HT_5A_R and 5-HT_6_R. It is speculated that the low levels of 5-HT_5A_R and 5-HT_6_R caused them to be undetectable. No significant difference in the extent of 5-HT receptor expression for each of the different receptor types was identified among the four types of muscle strip.

To the best of our knowledge, the present study is the first to identify 5-HT receptor mRNA and protein expression in the human LES. Although little information concerning the physiological and pharmacological effects of 5-HT receptors on the LES is available, the detection of 5-HT receptors in the present study supports the notion that the serotonergic system is an important modulator of esophageal motility. In the future, the development of specific ligands, as well as the use of gene deletion animal models such as knock-out mice for each 5-HT receptor, should allow precise evaluation of the physiological and pharmacological effects of specific 5-HT receptors in the LES.

## Figures and Tables

**Table I:** Primer sequences and expected product sizes for RT-PCR.

Gene	Primer pair sequence (sense/antisense)	Product size (bp)
5-HT_1A_R	5′-GGCGGCAACACTACTGGTAT-3′ 5′-AGCCAAGTGAGCGAGATGAG -3′	422
5-HT_2A_R	5′-ACTCGCCGATGATAACTTTGTCCT-3′ 5′-TGACGGCCATGATGTTTGTGAT-3′	359
5-HT_3A_R	5′-CCGGCGGCCCCTCTTCTAT-3′ 5′-GCAAAGTAGCCAGGCGATTCTCT-3′	448/352
5-HT_4_R	5′-GGCCTTCTACATCCCATTTCTCCT-3′ 5′-CTTCGGTAGCGCTCATCATCACA-3′	411
5-HT_5A_R	5′-CCCTTCTGCAAGTACCCCAG-3′ 5′-ATGACGTTGGAGACGCACTT-3′	522
5-HT_6_R	5′-CCGCCGGCCATGCTGAACG-3′ 5′-GCCCGACGCCACAAGGACAAAAG-3′	342
5-HT_7_R	5′-GCGCTGGCCGACCTCTC-3′ 5′-TCTTCCTGGCAGCCTTGTAAATCT-3′	436
β-actin	5′-GTGGGGCGCCCCAGGCACCA-3′ 5′-CTCCTTAATGTCACGCACGATTTC-3′	540

RT-PCR, reverse transcription-polymerase chain reaction; 5-HT, serotonin; R, receptor.
